# Exploring intensive care nurses’ team performance in a simulation-based emergency situation, − expert raters’ assessments versus self-assessments: an explorative study

**DOI:** 10.1186/s12912-014-0047-5

**Published:** 2014-12-17

**Authors:** Randi Ballangrud, Mona Persenius, Birgitta Hedelin, Marie Louise Hall-Lord

**Affiliations:** Department of Health Sciences, Faculty of Health, Science and Technology, Karlstad University, 651 88, Karlstad, Sweden; Department of Nursing, Faculty of Health, Care and Nursing, Gjøvik University College, Teknologivn. 22, 2815 Gjøvik, Norway

**Keywords:** Assessment, Emergency, Intensive care, Non-technical skills, Nursing, Patient safety, Simulation-based training, Team performance

## Abstract

**Background:**

Effective teamwork has proven to be crucial for providing safe care. The performance of emergencies in general and cardiac arrest situations in particular, has been criticized for primarily focusing on the individual’s technical skills and too little on the teams’ performance of non-technical skills. The aim of the study was to explore intensive care nurses’ team performance in a simulation-based emergency situation by using expert raters’ assessments and nurses’ self-assessments in relation to different intensive care specialties.

**Methods:**

The study used an explorative design based on laboratory high-fidelity simulation. Fifty-three registered nurses, who were allocated into 11 teams representing two intensive care specialties, participated in a videotaped simulation-based cardiac arrest setting. The expert raters used the Ottawa Crisis Resource Management Global Rating Scale and the first part of the Mayo High Performance Teamwork Scale to assess the teams’ performance. The registered nurses used the first part of the Mayo High Performance Teamwork Scale for their self-assessments, and the analyses used were Chi-square tests, Mann–Whitney *U* tests, Spearman’s rho and Intraclass Correlation Coefficient Type III.

**Results:**

The expert raters assessed the teams’ performance as either advanced novice or competent, with significant differences being found between the teams from different specialties. Significant differences were found between the expert raters’ assessments and the registered nurses’ self-assessments.

**Conclusions:**

Teams of registered nurses representing specialties with coronary patients exhibit a higher competence in non-technical skills compared to team performance regarding a simulated cardiac arrest. The use of expert raters’ assessments and registered nurses’ self-assessments are useful in raising awareness of team performance with regard to patient safety.

**Electronic supplementary material:**

The online version of this article (doi:10.1186/s12912-014-0047-5) contains supplementary material, which is available to authorized users.

## Background

Structuring work in health-care teams has been found to largely account for quality and safety improvements in patient care [1,2], and is shown as being crucial for patient safety in the intensive care unit (ICU). Failures in team processes such as communication, coordination or leadership have frequently been associated with patient safety incidents [1,3]. Effective teamwork in emergency situations is expected, although teamwork is not often evaluated or discussed on a regular basis in clinical practice [4]. Effective team training is recommended for those who are expected to work in teams [5], and nurses and physicians should conduct both disciplinary and interdisciplinary team training to increase patient safety. Simulation-based training is recommended today as a method to make health-care professionals aware of and understand the importance of teamwork and the aspects of team performance [6,7]. Moreover, simulation is seen as an effective method for assessing the ongoing competence of critical care nurses [8,9].

According to Flin et al. [10], teamwork consists of a number of elements such as supporting others, solving conflicts, exchanging information and coordinating activities, with one of the approaches used to minimize the effect of human error being to assist health-care teams in team performance training [11] by increasing individuals’ and teams’ competencies in non-technical skills (NTS) [12,13]. NTS are described as the “cognitive, social and personal resource skills that complement technical skills and contribute to safe and efficient task performance” [10]. According to Flin et al. [14], NTS generally include situational awareness, decision-making, communication, teamwork, leadership and the management of stress and fatigue. Team performance evaluation can be defined as, “the application of standard diagnostic measurement tools to assess the behaviors, cognitions and attitudes enacted by team members in relation to clearly operationalized criteria” [15]. NTS underpin the domain-specific competencies for the profession and team training programmes designed to increase the use of NTS, often referred to as crisis resource management (CRM) [16]. CRM aims to coordinate, utilize and apply all available resources to help optimize patient safety and outcome, as well as preventing errors and minimizing the negative consequences of errors that have already occurred. In addition to equipment, resources include all people involved with their abilities, attitudes, skills and limitations [17]. The principles for training teams to cope with stressful situations and error management were developed by the airline industry [18] and transferred to health care by Gaba et al. [19], who adapted the use of human patient simulators into the CRM programme. With regard to CRM programmes, NTS are often termed as CRM skills [20,21].

Reader et al. [22] identified that a large proportion of the contributory factors underlying critical incidents in the ICU could be attributed to poor NTS in terms of communication, leadership, coordination and decision making. Furthermore, data from an ICU incident-reporting system showed that team communication failure led to patient harm [23,24], while critical incident studies have indicated the importance of team leadership for guiding the way in which ICU team members interact and coordinate [24,25]. With regard to cardiopulmonary resuscitation (CPR) situations, simulation-based research has demonstrated that an absence of leadership and task distribution was associated with poor interdisciplinary team performance [26]. However, hierarchies have been identified as barriers to a team’s action [27,28], while interdisciplinary teamwork is seen as one of the key processes in the safe delivery of patient care [5]. Nonetheless, by observing the simulation-based interdisciplinary interaction of nurses in obstetric and neonatal emergencies, Miller et al. [29] found a need for improvement with regard to nurses’ NTS necessary for their contribution to high-reliability teams.

Team performance measurement is important in order to verify team effectiveness and to identify strengths and weaknesses as a basis for further training and continuing education to help achieve a safe and effective patient care [30,31]. To identify team performance, the evaluation may include both observational expert assessments and team self-assessment [15], though research has documented an incongruity between self-assessment compared with the observed measures of NTS competence [32,33]. Nurses who work in different intensive care specialties with somewhat varied categories of critically ill patients are involved in emergency situations such as cardiac arrests in both real- and training situations. However, the CPR performing situations have been criticized for mainly focusing on the individual’s technical skills and too little on the team’s NTS performance [26-28]. There are few available studies that focus on ICU nurses’ NTS competence in emergencies team performance. Therefore, the aim of this study was to explore intensive care nurses’ team performance in a simulation-based emergency situation by using expert raters’ assessments and nurses’ self-assessments in relation to different intensive care specialties.

## Methods

### Design

The study used an explorative design based on laboratory high-fidelity simulation.

### Setting

The study was conducted in a simulation centre at a university college in Norway, in a laboratory created as an ICU environment and with the use of a high-fidelity human patient simulator.

### Participants

A convenience sample of 53 registered nurses (RNs) was recruited from seven ICUs in four hospitals within one hospital trust (Table 1).

**Table 1:** **Characteristics of the sample (11 teams, n = 53 RNs)**

**Demographic information**	**Subcategory**	**Subgroups**		
			**Mean (SD)**	**n (%)**
Specialties (n = 2)		G-ICU^1^ (5 teams)		26 (49.1)
		M-ICU^2^ (6 teams)		27 (50.9)
Age				
		G-ICU	46.69 (6.98)	
		M-ICU	43.59 (8.04)	
Gender	Female			
		G-ICU		24 (92.3)
		M-ICU		24 (88.9)
	Male			
		G-ICU		2 (7.7)
		M-ICU		3 (11.1)
Education level	Postgraduate			
		G-ICU		26 (100.0)
		M-ICU		24 (88.9)
Years as PG^3^- RN (n = 50)				
		G-ICU	14.38 (8.90)	
		M-ICU	7.28 (8.62)	
Years at unit				
		G-ICU	14.73 (8.90	
		M-ICU	8.52 (6.86)	

^1^G-ICU = General intensive care unit, ^2^M-ICU = Medical intensive care unit, ^3^PG = Postgraduate.

The RNs worked in two specialties: (i) general intensive care units (G-ICU) (n = 26 RNs) with surgical and medical patients, usually without coronary patients, and (ii) medical intensive care units (M-ICU) (n = 27 RNs) with coronary, medical and in one unit also surgical patients. Participation in the study occurred during the RNs’ scheduled work time and the RNs who wanted to participate signed up on a list. Each team consisted of four to six RNs from same units who knew each other and had worked together. The unit nurse/manager allocated the RNs into teams with regard to their work schedules and the staffing resources to ensure safe care at the unit. A total of 11 teams from the G-ICU (five teams) and M-ICU (six teams) participated.

### Simulation-based team training programme and scenario

The cardiac arrest scenario chosen for this study, was a part of a simulation-based team training programme which comprised two half days for each team, and was developed by the research group.

On the first half day of the programme, the RNs received information about the topic of the simulation scenario cases, the CRM learning objectives and recommendations about relevant literature to read. Furthermore, the RNs participated in theory inputs concerning patient safety in the ICU, NTS with a focus on the key CRM points by Rall and Gaba [34] and the use of simulation for training.

The other half day of the programme that referred to conducting two simulation scenarios, was carried out for one team at a time two to four weeks after the first day. In addition to the scenario which involved a patient undergoing cardiac arrest (reported in this study), a scenario of a receipt of a trauma patient was conducted. The two simulation scenarios were carried out in varying order for each team within each specialty. Two trained simulator instructors were responsible for the implementation of the scenario, one for facilitating the simulation and the other for performing the operation of the mannequin software. Three different scenario roles were randomly assigned among the team members by the facilitator: one as a patient charge nurse (leader), one as the charge nurse’s assistant (assistant) and the third with the responsibility for the patient in the neighboring bed (helper). The RNs also had the opportunity to call a physician (acted out by the facilitator), who gave the RNs medical directives for the handling of the emergency over the phone. The rest of the team members were placed in another room and observed the team performance on a screen, with the videotaped scenario lasting approximately 12–15 minutes.

#### Cardiac Arrest simulation scenario

A patient (simulator), 55-year-old man arrived at the ICU three days ago with a diagnosis of cardiac arrest. He has been through an effective implementation of 24 hours of therapeutic hypothermia treatment and has been extubated for some few hours. He is calling for help and the RNs that had just arrived on duty enter the patient room. The patient is suffering from chest pain and is restless and anxious. A while after the RNs entered the patient’s room, and the patient suffered cardiac arrest due to ventricular tachycardia displayed on the monitor. With the onset of cardiac arrest, the patient closed his eyes, ceased to speak and to breathe, and his pulse was no longer palpable. Cardiopulmonary resuscitation was expected to be initiated by the RNs. Regardless of the measures taken, the patient stayed in cardiac arrest for many minutes. Thereafter, ROSC could be achieved by defibrillation.

#### Team training objectives

- Practicing an organized and effective problem solving
- Adapts leadership skills based on the team composition and situation
- Communicates clearly and concisely
- Utilize available resources
- Constantly reassesses and reevaluates situation

### Data collection

#### Procedure

The data collection took place from April 2009 to May 2010, and immediately after completing the scenario all the RNs in the team conducted a team performance self–assessment using Mayo High Performance Teamwork Scale (MHPTS) (items 1–8) [20]. After all teams had completed the simulation-based team training programme, two trained expert raters, who had no contact with each other, individually viewed each videotaped scenario for all 11 teams and assessed their team performance by using Ottawa Crisis Resource Management Global Rating Scale (Ottawa GRS) [21] and MHPTS (items 1–8). The raters were postgraduate (PG) ICU RNs chosen for their expertise as CRM simulation instructors. One-day rater training organized by the researcher and the simulation instructors was conducted in order to standardize the evaluation of team performance and to become familiar with the instruments. Video files from a pilot-testing of the scenarios, with teams not involved in the study performing the scenario, were used in the training of the raters. After watching the video, each rater individually rated the video files of different teams by using the Ottawa GRS and MHPTS, and then together they reconsidered their rating in order to develop a common understanding of the team’s performance.

### Instruments

MHPTS [20] provides a brief, reliable and practical measure of NTS that can be used by participants as a self-assessment of CRM training, in which they reflect on and evaluate their team performance. The scale is also recommended for comparisons between self-assessment and expert assessment [20]. Each item is eligible for a 0-, 1- or 2 point assessment: 0 = never or rarely, 1 = inconsistently and 2 = consistently. The first part of MHPTS (items 1–8) was used because the items were judged as being consistent with the simulation scenario.

#### MHPTS assessment criteria

1. A leader is clearly recognized by all members.
2. The team leader assures maintenance of an appropriate balance between command authority and team member participation.
3. Each team member demonstrates a clear understanding of his or her role.
4. The team prompts each other to attend all significant clinical indicators throughout the procedure/intervention.
5. When team members are actively involved with the patient, they verbalize their activities aloud.
6. Team members repeat back or paraphrase instructions and clarifications to indicate that they heard them correctly.
7. Team members refer to established protocols and checklists for the procedure/intervention.
8. All members of the team are appropriately involved and participate in the activity.

Ottawa GRS [21] provides an observational expert rating scale which consists of six categories: one overall CRM performance category (Overall-CRM) and five subsets of CRM skills (NTS) categories, including assessment criteria regarding leadership, problem solving, situational awareness, resource utilization and communication. All categories used a seven-point adjective scale, using a rating from 1-2 = novice (all CRM skills requiring a significant improvement), 3-4 = advanced novice (many CRM skills requiring a moderate improvement), 5-6 = competent (most CRM skills requiring minor improvement) and 7 = clearly superior (few, if any, CRM skills which only require a minor improvement).

#### Ottawa CRM skills categories with assessment criteria

*Leader ship:*

- Stay calm and in control during crisis
- Prompt and firm decision-making
- Maintains global perspective (“Big picture”)

*Problem solving:*

- Organized and efficient problem solving approach (ABC’s)
- Quick in implementation (Concurrent management)
- Considers alternatives during crisis

*Situation awareness:*

- Avoid fixation error
- Reassess and re-evaluates situation constantly.
- Anticipates likely events

*Resource utilization:*

- Calls for help appropriately
- Utilizes resources at hand appropriately
- Prioritizes tasks appropriately

*Communication:*

- Communicate clearly and concisely
- Uses directed verbal/non-verbal communication
- Listen to team input

In addition, seven background questions on age, gender, education level, type of unit, years as RN and postgraduate RN and years on present unit were included.

The MHPTS and Ottawa GRS were translated into Norwegian using a “back translation” [35], and the instruments were tested for both face- and content validity by an expert group of simulation instructors, with only minor linguistic adjustments being required.

### Ethical considerations

This study was approved by the Norwegian Social Data Services as well as being approved by the hospital’s administrative heads, and the study was conducted according to the Ethical Guidelines for Nursing Research in the Nordic Countries [36]. Information, and an invitation to participate in the study were given to the RNs in both written and oral form, and referred to the principle of autonomy addressed by voluntariness, informed consent and the right to withdraw from the research project at any time, as well as confidentiality in terms of making data anonymous and assurance that the videotapes would been deleted at the end of the project. The RNs written consent was obtained.

### Data analyses

IBM SPSS Statistics 19 was used to analyse the data, with descriptive statistics presenting the frequencies, percentages, means and standard deviations. The units of analyses of team performance were the team score for the expert raters’ assessments and the RNs’ self-assessments, respectively. Chi-square tests were used to compare the two specialties with regard to the RNs’ background in general and with regard to the three different scenario roles. The expert raters’ assessments were calculated by adding each rater’s score for each team, and then dividing by two. Mann–Whitney *U* tests were conducted to compare the differences between the teams from two types of specialties (G-ICU, M-ICU) with regard to the Ottawa GRS CRM categories and the MHPTS items. The RNs’ self-assessments were calculated by adding each team member’s score for the team, and then dividing the score by the number of team members. Mann–Whitney *U* tests were conducted to compare the differences between the teams from two types of specialties (G-ICU, M-ICU) concerning the MHPTS items. Additionally, Mann–Whitney *U* tests were used to compare the differences between the expert raters’ assessments and the RNs’ self-assessments of the MHPTS items. Correlational analyses with Spearman’s rho were used to explore the relationship between the expert raters’ assessments and the RNs’ self-assessments of the MHPTS items. A two-tailed significance level of p < .05 was used for all tests.

Intraclass Correlation Coefficient (ICC) Type III was used to assess inter-rater reliability regarding Ottawa GRS CRM categories. ICC coefficients > .75 indicate a good reliability, with coefficients from .50 -.75 suggesting moderate reliability and values < .50 representing poor reliability [37]. This study showed an ICC ranging from .667-.854 (moderate - good reliability) (Table 2).

**Table 2:** **Ottawa GRS interrater reliability intraclass correlation (ICC)**

**Ottawa GRS categories**	**Type III ICC** ^**1**^
Overall	.729
Leadership	.854
Problem solving	.742
Situation awareness	.667
Resource utilization	.735
Communication	.788

^1^Two-way mixed effects model.

Percentage agreements were conducted in relation to MHPTS, and in this study the percentage of agreements between raters was 60%.

## Results

Most of the 53 participants were women (91%) and were post-graduates (94%). Comparisons between the two specialties’ background showed significant differences with regard to “years as PG-RNs” (χ^2^ (2) =12.53, *p* = .002), and “years at unit” (χ^2^ (2) =7.32, *p* = .026), with the RNs from the G-ICU with the longest experience as PG-RNs and most years at unit. In relation to scenario roles, significant differences were found with regard to the helper role in “age” (χ^2^ (2) = 7.71, *p =* .013) and in “years as PG-RNs” (χ^2^ (2) =7.81, *p* = .008), with helpers in teams representing G-ICU with a higher age and longer experience as PG-RNs.

The expert raters’ assessments of the 11 teams’ performance in relation to the different specialties of unit based on Ottawa GRS are shown in Table 3.

**Table 3:** **Expert raters’ assessments in relation to types of specialties**

**Ottawa GRS CRM categories**	**G-ICU** ^**1**^ **(n = 5)**	**M-ICU** ^**2**^ **(n = 6)**	**Mann–Whitney** ***U*** **tests**	
	**mean (SD)**	**mean (SD)**	**Z**	***p***
Overall performance	3.70 (1.04)	5.92 (0.58)	−2.666	**.008**
Leadership skills	3.80 (0.67)	5.83 (0.52)	−2.777	**.005**
Problem solving skills	3.30 (0.67)	5.83 (0.61)	−2.764	**.006**
Situational awareness skills	3.70 (0.84)	5.83 (0.61)	−2.764	**.006**
Resource utilization skills	3.60 (0.82)	5.83 (0.26)	−2.844	**.004**
Communication skills	3.50 (0.71)	5.58 (0.38)	−2.796	**.005**

Rating: 1-2 = novice, 3-4 = advanced novice, 5-6 = competent and 7 = clearly superior.

^1^G-ICU = General intensive care unit. ^2^M-ICU = Medical intensive care unit.

The teams’ effectiveness ranged from “advanced novice” to “competent”, and there were significant differences between teams from different specialties in all six of the Ottawa GRS categories. Moreover, the M-ICU teams achieved higher scores than the G-ICU teams.

The expert raters’ assessments and the RNs’ self-assessments of team performance in relation to the different specialties, as well as the comparisons between expert raters’ assessments and RNs’ self-assessments based on MHPTS, are shown in Table 4.

**Table 4:** **Expert raters’ assessments and RNs’ self-assessments in relation to types of specialties**

			**Expert raters’ assessments**			**RNs’ self-assessments**			**Comparisons of expert raters’ and RNs’ self-assessments**	
**MHPTS Item**		**Specialties**		**Mann–Whitney** ***U*** **tests**			**Mann–Whitney** ***U*** **tests**		**Mann–Whitney** ***U*** **tests**	
			**Mean (SD)**	**Z**	***p***	**Mean (SD)**	**Z**	***p***	**Z**	***p***
1	A leader is clearly recognized by all members	G-ICU^1^	0.90 (0.55)	−2.191	**.028**	1.45 (0.19)	−1.287	.198	−1.954	.051
		M-ICU^2^	1.67 (0.41)			1.22 (0.31)			−2.021	**.044**
2	The team leader assures maintenance of an appropriate balance between command authority and team member participation	G-ICU	0.80 (0.57)	−2.175	**.030**	1.45 (0.19)	*-.463*	.644	−1.972	**.049**
		M-ICU	1.58 (0.38)			1.38 (0.22)			−1.366	.172
3	Each team member demonstrates a clear understanding of his or her role	G-ICU	1.00 (0.35)	−2.063	**.039**	1.46 (0.89)	-.835	.404	−2.010	**.044**
		M-ICU	1.50 (0.32)			1.49 (0.39)			-.337	.736
4	The team prompts each other to attend all significant clinical indicators throughout the procedure/intervention	G-ICU	0.70 (0.45)	−2.837	**.005**	1.26 (0.30)	−1.851	.064	−1.954	.051
		M-ICU	1.83 (0.26)			1.63 (0.18)			−2.037	**.042**
5	When team members are actively involved with the patient, they verbalize their activities aloud	G-ICU	0.80 (0.27)	−2.844	**.004**	1.33 (0.21)	−1.038	.299	−1.496	.135
		M-ICU	1.83 (0.26)			1.42 (0.20)			−2.024	.**043**
6	Team members repeat back or paraphrase instructions and clarifications to indicate that they heard them correctly	G-ICU	0.50 (0.35)	−2.837	**.005**	1.07 (0.29)	−1.116	.264	−1.775	.076
		M-ICU	1.83 (0.26)			1.33 (0.31)			−1.677	.094
7	Team members refer to established protocols and checklists for the procedure/intervention	G-ICU	0.40 (0.22)	−2.837	**.005**	0.74 (0.19)	−2.114	**.035**	-.804	.421
		M-ICU	1.58 (0.49)			1.27( 0.40)			−1.181	.238
8	All members of the team are appropriately involved and participate in the activity	G-ICU	1.20 (0.27)	−2.515	**.017**	1.53 (0.29)	−1.864	.062	−1.206	.381
		M-ICU	1.83 (0.26)			1.84 (0.12)			-.692	.489

Rating: 0 = never or rarely, 1 = inconsistently, 2 = consistently. ^1^G-ICU = General intensive care unit, ^2^M-ICU = Medical intensive care unit.

Bold numbers reflect significance level of *p* < .05.

The expert raters’ assessments demonstrated significant differences between the ICU teams for all eight MHPTS items, with the G-ICU teams having lower scores than the teams from the M-ICU. One significant difference was found between different intensive care specialties with regard to the RNs’ self-assessments. RNs’ scores for each team are shown in Additional file 1. Additionally, there were significant differences between the expert raters’ assessments and the RNs’ self-assessments on five items, with the teams from G-ICU having higher scores than the expert raters on two items and the teams from M-ICU having lower score than expert raters on three items. Correlations between expert raters’ assessments and the RNs’ self-assessments of the eight items demonstrated for G-ICU a variation from r = .00 to .92 and for M-ICU from r = −.11 to .50. Of a total of 16 analyses 13 showed positive correlations, with one significant correlation for G-ICU with regard to item 1 (r = .92, *p =* .028). No correlation was found on item 5 for both G-ICU and M-ICU, and the negative correlation was found on item 8 for G-ICU.

## Discussion

The main result of the study showed that the expert raters’ assessment exhibited a variation in the intensive care nurses’ team performance, as the teams from the M-ICU were given higher scores by the expert raters in regard to both the Ottawa GRS six categories and the MHPTS items. According to the Ottawa GRS criteria, the M-ICU was assessed as competent, which means that most CRM skills required some minor improvements. However, the G-ICU teams were assessed as being “advanced novice”, meaning that many CRM skills required some moderate improvement. Even so, none of the teams were assessed as being clearly superior. However, significant differences were found between the expert raters and the RNs’ self-assessment of their team’s performance according to MHPTS. To help ensure quality and patient safety, all RNs working in ICUs should possess the knowledge, skills and attitudes necessary to be an effective team member in an emergency situation, and the quality of care is no better than the quality created on the sharp end of care [38]. Team training has been found to lead to safety culture changes [39] and the M-ICUs with coronary patients have a long tradition of training CPR with a focus on technical skills [40], which may also have an influence on the team performance concerning NTS.

### RNs team performance

Team leadership refers to how to influence team performance by facilitating team problem solving through cognitive processes, coordination processes and the team’s collective motivation and behaviour [41]. In emergencies generally, and in cardiac arrest situations in particular, the behaviour of the first responders is crucial, and the first-responding RN should take the leadership role [42]. RNs from the M-ICU representing units with coronary patients may have experienced cardiac arrest situations in real- and training situations to a greater extent, and were therefore more aware of the leader’s role. Conversely, the RNs from G-ICU had the longest experience as PG-RNs and most years at unit. This may indicate the importance of regular training. A lack of knowledge of procedure or guidelines may result in low leadership performance [43], and research has shown that well-trained, first-reacting nurses have successfully taken leadership roles in CPR [44,45]. It is important that RNs stay in the leadership role until others with more leadership experience appear. Team effectiveness regarding CPR is found to be most optimal with a leader who demonstrates immediate directive leadership behaviour [46,47]. Teamwork may be affected by the institutional culture, and RNs from G-ICU may be less comfortable being a leader in a cardiac arrest situation. Still, Andersen et al. [27] found that the RNs’ cultural and professional role inhibited them from participating in decision making in the CPR despite being certified as Advanced Life Support instructors, even though nurses are responsible for decision making in emergencies [48]. According to Künzle et al. [43], an inadequate leadership behaviour in critical care teams could be due to a lack of leadership training; it is therefore important that all RNs in ICUs receive this training to help improve patient safety. RNs often embrace CRM training because it results in a greater respect and an improved quality of work life by flattening hierarchies of authority through team members’ shared responsibility for patient safety [49].

The teams from the M-ICU with the most experience with coronary patients achieved higher scores compared to the G-ICU with regard to problem solving in both the experts’-and the RNs’ self-assessments. However, research demonstrates that a failure to transform theoretical knowledge into effective team activity during CPR appears to be a major problem [26,50], which is a result that highlights the importance of training team performance. Thomas et al. [51] found that nurses who received team training as part of their neonatal resuscitation training were generally shown to display more teamwork-related behaviours than those who did not receive such training. Skills identified as being the most improved during patient emergencies after followed up simulation-based team training were found to be the ability to respond in a systematic way, handover to the emergency team and airway management [52].

All teams demonstrated positive results regarding the MHPTS’s resource utilization skills as assessed by both the expert raters and the RNs. The RNs from the G-ICU assessed themselves significantly higher than the experts in the items “each team demonstrated a clear understanding of his or her role”. One reason for this more positive assessment may be that the RNs in the scenario were assigned different roles by the facilitator, which helped them to clarify their function in the team. However, patient safety climate studies confirm that RNs in ICUs have positive perceptions of the teamwork within the unit which includes, e.g. supporting others and coordinating activities [53-55], which may also influence the team’s resource utilization skills.

A similar picture emerged with the M-ICU teams, with higher scores than the G-ICU teams with regard to communication skills, which may be a result of RN teams from the M-ICU being more familiar with the CPR guidelines. This resonates with “shared mental model” literature, in which teams communicate and coordinate more effectively when members form a shared mental model for goals, tasks and team member roles and responsibilities [56]. As one of the central components of health-care teamwork, communication has been identified for several years as a central root cause of sentinel events [57]. Tools such as SBAR (situation, background, assessment, recommendation), which provide a common and predictable structure to the communication, as well as a “critical language” that gets everyone to stop and listen, have been found to be effective and essential for safe care in complex environments [58]. Furthermore, “closed-loop communication”, with the verbal confirmation of orders and messages for ensuring that sent communications are heard and accurately understood, is recommended [56]. The implementation of standardized communication tools and techniques may improve team performance in an emergency, thereby exerting a positive impact on patient safety, which should be recommended to be adopted for team training.

### Expert rater assessment vs. self-assessment

The present study included both self-assessments of team performance and video-based analyses by expert raters to study RNs’ team performance. Together with direct observation, these are in accordance with the most up-to-date measures of team performance in health care [31]. Video-based analyses offer advantages since they allow for a precise analysis and repeated review by multiple experts [31], and the observational measures may capture the behaviour that teams actually engage in, although observers cannot assess all of the implicit components of teamwork [15]. Situational awareness skills represent implicit components of teamwork, and self-assessments are of great importance. The RNs from G-ICU assessed themselves as generally higher than the expert raters, and significant differences were shown on the two items referring to leadership and resource utilization. On the other hand, the RNs from M-ICU assessed themselves generally lower than the experts, with significant differences on the three items referring to leadership, situational awareness and communication skills. An incongruity between RNs’ self-assessment and the experts’ assessment may indicate that RNs both overestimated and underestimated their NTS competence. Although the correlations between self-assessment and the experts’ assessment had positive directions in 13 of 16 analyses, only one significant relationship was found. Moreover, no correlations (item 5) and a negative correlation (item 8) may also show an incongruity assessment among the RNs. Arora et al. [33] found a strong correlation between surgeons’ self-assessments and expert assessments with regard to technical skills, though no significant correlations were found with regard to NTS. Davis et al.’s [32] systematic review of the accuracy of physicians’ self-assessments compared with observed expert measures of competence documented that in a majority of the relevant studies, physicians did not appear to accurately self-assess. Our results indicate a tendency of poor performers to overestimate their performance while high performers underestimate theirs. Kruger and Dunning [59] studied the calibration of subjects carrying out various tasks such as logical reasoning, grammar and humour. They demonstrated that participants who were in the lowest-scoring quartile greatly overestimated their abilities; those in the middle quartiles were generally accurate in their self-assessment; whereas participants who had the highest scores underestimated their abilities. These tendencies were confirmed by Hodges et al. [60] studying family medicine residents who interviewed a standardized patient in a difficult “telling bad news” scenario.

While self-report measures have their limitations, they offer a means for assessing unobservable components of team performance, which are no less important than observable components pertaining to training [15]. Different approaches in evaluating team performance have both their strengths and weaknesses [15,31], and a combined approach can help to obtain a more complete picture of the complexity of team performance, while also providing a method to guide learning through systematic feedback [15].

### Limitations

A limitation of this study was the small number of ICU teams; therefore, the generalizability of the results must be interpreted with caution. Additionally, the result of the RNs’ team performance may be influenced by participants who were initially positive to the use of simulation-based training, and on this basis agreed to participate. Even though simulation studies have many opportunities and advantages, there are also limitations, and although behaviour patterns in simulation are similar to those in real-life situations, the transfer of knowledge from the simulator to real-patient situations has not been clearly confirmed [61]. Although rater training was conducted in order to standardize the evaluation of teamwork performance, some moderate ICC scores were demonstrated regarding the Ottawa GRS. Moreover, a 60% agreement was found for the MHPTS rating of the teams’ performance. The complexities of team performance pose a challenge, and measurement and evaluation have weaknesses due to responsible biases of individual raters [15,30], which may influence the achievement of a high reliability.

## Conclusion

Simulation-based team performance measurement among ICU nurses revealed a variation in team performance in a simulated cardiac arrest emergency situation. RNs’ team performance required minor to moderate improvements, and the RNs with the most experience from coronary patients showed the best team performance. The use of expert raters’ assessments and RNs’ self-assessment is useful in raising awareness of team performance with regard to patient safety.

One important implication for practice is that the results contribute to a focus on RNs’ knowledge, skills and attitudes regarding team performance in order to ensure quality and patient safety in emergency patient situations in the ICU. Further research in terms of the RNs’ contribution of NTS is needed, while it will also be of great interest to investigate the ICU RNs’ perceptions on the use of simulations for team training in promoting patient safety in the ICU.

## Additional file

Additional file 1:
**RNs’ team score in relation to type of specialties.**

## Abbreviations

CPR: Cardiopulmonary resuscitation
CRM: Crisis recourse management
G-ICU: General intensive care units
ICU: Intensive care unit
ICC: Intraclass correlation coefficient
M-ICU: Medical intensive care units
MHPTS: Mayo high performance teamwork scale
NTS: Non-technical skills
Ottawa GRS: Ottawa crisis resource management global rating scale
RN: Registered nurse
SBAR: Situation, background, assessment, recommendation
