# Supplementary material for: Exploring intensive care nurses’ team performance in a simulation-based emergency situation, − expert raters’ assessments versus self-assessments: an explorative study
Source: BMC Nurs. 2014 Dec 17;13:47. doi: 10.1186/s12912-014-0047-5 (PMC4299298; doi:10.1186/s12912-014-0047-5)
Supplement: Additional file 1: — RNs’ team score in relation to type of specialties. [file 12912_2014_47_MOESM1_ESM.doc]

**Additional file 1: RNs’ t**eam score in relation to type of specialties

|  |  | **G-ICU^1^** | | | | | **M-ICU^2^** | | | | | |
| --- | --- | --- | --- | --- | --- | --- | --- | --- | --- | --- | --- | --- |
|  | Team | 1 | 2 | 3 | 4 | 5 | 6 | 7 | 8 | 9 | 10 | 11 |
|  | **MHPTS items** | mean (SD) | mean (SD) | mean (SD) | mean (SD) | mean (SD) | mean (SD) | mean (SD) | mean (SD) | mean (SD) | mean (SD) | mean (SD) |
| 1 | A leader is clearly recognized by all members | 1.40 (0.55) | 1.67 (0.52) | 1.20 (0.45) | 1.60 (0.55) | 1.40 (0.55) | 1.20 (0.45) | 1.60 (0.55) | 1.00 (0.00) | 0.75 (0.50) | 1.25 (0.50) | 1.50 (0.57) |
| 2 | The team leader assures maintenance of an appropriate  balance between command authority and team member  participation | 1.40  (0.55) | 1.67 (0.52) | 1.40 (0.55) | 1.60 (0.55) | 1.20 (0.45) | 1.40 (0.55) | 1.60 (0.55) | 1.00 (0.71) | 1.25 (0.96) | 1.50 (0.58) | 1.50 (0.57) |
| 3 | Each team member demonstrates a clear understanding  of his or her role | 1.40 (0.55) | 1.50 (0.55) | 1.40 (0.55) | 1.40 (0.55) | 1.60 (0.55) | 1.60 (0.55) | 1.80 (0.45) | 1.80 (0.45) | 1.00 (0.00) | 1.00 (1.00) | 1.75 (0.50) |
| 4 | The team prompts each other to attend all significant  clinical indicators throughout the procedure / intervention | 1.00 (0.00) | 1.50 (0.55) | 1.00 (0.00) | 1.20 (0.45) | 1.60 (0.55) | 1.80 (0.45) | 1.40 (0.55) | 1.80 (0.45) | 1.50 (0.58) | 1.50 (0.58) | 1.75 (0.50) |
| 5 | When team members are actively involved with the  patient, they verbalize their activities aloud | 1.20 (0.45) | 1.67 (0.52) | 1.40 (0.55) | 1.20 (0.45) | 1.20 (0.45) | 1.40 (0.55) | 1.20 (0.45) | 1.40 (0.55) | 1.50 (0.58) | 1.75 (0.50) | 1.25 (0.50) |
| 6 | Team members repeat back or paraphrase instructions and clarifications to indicate that they heard them correctly | 1.00 (0.00) | 1.33 (0.52) | 0.60 (0.55) | 1.20 (0.45) | 1.20 (0.45) | 1.40 (0.55) | 1.60 (0.55) | 1.20 (0.45) | 1.75 (0.50) | 1.00 (0.82) | 1.00 (0.00) |
| 7 | Team members refer to established protocols and checklists for the procedure/ intervention. | 0.60 (0.55) | 1.00 (0.63) | 0.80 (0.45) | 0.80 (0.84) | 0.50 (0.58) | 0.60 (0.55) | 1.80 (0.45) | 1.20 (0.84) | 1.25 (0.50) | 1.25 (0.96) | 1.50 (0.57) |
| 8 | All members of the team are  appropriately involved and participate in the activity | 1.60 (0.55) | 2.00 (0.00) | 1.40 (0.55) | 1.25 (0.50) | 1.40 (0.55) | 1.80 (0.45) | 2.00 (0.00) | 2.00 (0.00) | 1.75 (0.50) | 1.75 (0.50) | 1.75 (0.50) |

Rating: 0=never or rarely, 1=inconsistently, 2=consistently. ^1^G-ICU=General intensive care unit, ^2^ M-ICU= Medical intensive care unit
